# The tree-based pipeline optimization tool: Tackling biomedical research problems with genetic programming and automated machine learning

**DOI:** 10.1016/j.patter.2025.101314

**Published:** 2025-07-11

**Authors:** Jose Guadalupe Hernandez, Anil Kumar Saini, Attri Ghosh, Jason H. Moore

**Affiliations:** 1 Cedars-Sinai Medical Center, Los Angeles, CA, USA

**Keywords:** TPOT, automated machine learning, genetic programming, computational biomedicine, pipeline optimization, evolutionary computation, Pareto optimization

## Abstract

The tree-based pipeline optimization tool (TPOT) is one of the earliest automated machine learning (ML) frameworks developed for optimizing ML pipelines, with an emphasis on addressing the complexities of biomedical research. TPOT uses genetic programming to explore a diverse space of pipeline structures and hyperparameter configurations in search of optimal pipelines. Here, we provide a comparative overview of the conceptual similarities and implementation differences between the previous and latest versions of TPOT, focusing on two key aspects: (1) the representation of ML pipelines and (2) the underlying algorithm driving pipeline optimization. We also highlight TPOT’s application across various medical and healthcare domains, including disease diagnosis, adverse outcome forecasting, and genetic analysis. Additionally, we propose future directions for enhancing TPOT by integrating contemporary ML techniques and recent advancements in evolutionary computation.

## Highlights

• TPOT is an AutoML tool that optimizes ML pipelines using genetic programming
• TPOT simplifies pipeline design, aiding biomedical research and healthcare tasks
• TPOT has been applied in disease diagnosis, genetic analysis, and medical outcome prediction

## Teaser

The tree-based pipeline optimization tool (TPOT) is an automated machine learning framework that uses genetic programming to optimize ML pipelines, particularly for biomedical research. It simplifies pipeline design and has been applied in disease diagnosis, genetic analysis, and healthcare outcome prediction. In this resource, the authors describe the improvements to the latest version and highlight future enhancements to incorporate cutting-edge ML techniques and evolutionary strategies.

## Introduction

Machine learning (ML) has been successfully integrated into medicine and healthcare, leading to improved patient outcomes while reducing overall costs.^1^^,^^2^^,^^3^ Notable successes include advancements in precision medicine,^4^^,^^5^ drug discovery,^6^^,^^7^^,^^8^ and disease diagnosis.^9^^,^^10^^,^^11^^,^^12^ The increasing availability of computational resources and the rapid accumulation of medical data create an optimal environment for ML to advance and drive innovation in healthcare. However, these factors alone do not necessarily simplify the implementation of ML in medical and healthcare applications. One difficulty arises from the data themselves. For example, while there is no shortage of medical data, the data exist in various formats,^3^ such as electronic health records (EHRs), genomic data, medical images, and clinical notes, each requiring a specialized ML approach for effective analysis and application. Furthermore, there is no guarantee that the available data accurately capture underlying patterns, as EHR data often contain inconsistencies and inaccuracies,^13^^,^^14^ making it challenging for ML approaches to learn these patterns.

The second difficulty arises from using the dataset to answer a research question. Before implementing ML in healthcare or medical applications, a clearly defined and testable research question must first be established.^15^ However, formulating an effective research question for this purpose is challenging because of the difficulty in defining a measure of success. For example, highly accurate predictive models might be preferable but not at the cost of making biased predictions for certain communities. Nevertheless, once a well-defined research question is established and a reliable dataset is identified, researchers can focus on developing an ML approach to address the question. Typically, researchers are challenged with engineering an ML pipeline consisting of one or more ML methods tailored to the specific task, such as classification or regression. For example, a pipeline may begin with feature engineering, where raw features are transformed, followed by feature selection, which identifies a subset of features from both the original and transformed features. The selected features are then fed into a classifier or regressor for model training. While this example outlines a simple pipeline with the general purpose of each stage, in the real world, one must discover the optimal pipeline configuration or structure, which often requires software engineering expertise and time-consuming analysis. Once the pipeline structure has been decided, researchers must still explore a vast search space of ML methods for each stage while simultaneously optimizing the hyperparameters associated with each chosen approach. Additionally, domain expertise may be necessary to guide the construction and optimization of the ML pipeline, ensuring its suitability for the specific problem.^1^^,^^15^ Integrating ML into medical and healthcare applications presents significant challenges; however, the benefits make the effort worthwhile.

Automated ML (AutoML) for ML pipeline optimization aims to automatically determine the optimal combination of ML methods for a pipeline while simultaneously tuning their hyperparameters.^16^ Before the development of AutoML, selecting the optimal ML methods within a pipeline and tuning their hyperparameters were treated as separate tasks. All AutoML systems can be characterized by three key aspects^17^: (1) the pipeline search space, which defines the set of possible ML pipelines; (2) the optimization strategy used to explore and refine pipelines; and (3) the evaluation strategy used to assess the generalizability of a given pipeline. Ideally, an AutoML system should define a search space that includes the optimal pipeline while using an effective and efficient optimization strategy to identify it based on the results produced by the chosen evaluation strategy. Some of the most commonly used optimization strategies in AutoML include Bayesian optimization,^18^^,^^19^ evolutionary algorithms,^20^^,^^21^^,^^22^ and reinforcement learning,^23^^,^^24^ each with its own unique advantages and limitations. Interestingly, the “no free lunch” theorem^25^ asserts that no single optimization technique can consistently outperform all others across all possible problems. This theorem highlights the necessity of exploring multiple approaches for the complex task of pipeline optimization.

The tree-based pipeline optimization tool (TPOT)^26^ is one of the first AutoML frameworks developed for ML pipeline optimization and the first to incorporate genetic programming (GP)^27^ as its optimization strategy. GP is a population-based optimization strategy inspired by biological evolution. Note that we will use TPOT to refer to the general package and TPOT1 and TPOT2 to distinguish between the two distinct implementations wherever applicable. TPOT is an open-source project available on GitHub (https://github.com/EpistasisLab/tpot), gaining popularity as it nears 10,000 stars and 2,000 forks. The development of TPOT was motivated by two fundamental challenges in ML. The first challenge was the identification of optimal ML pipelines for any given dataset. Requirements for methodological development were an algorithm that could explore a diverse set of pipeline architectures and configurations using open-source ML tools. GP was an ideal choice for this task, given its ability to represent ML pipelines as computer programs and its parallel search and optimization features, which include multiobjective methods, such as Pareto optimization,^28^^,^^29^ that are often needed for biomedical problems where users want pipelines that satisfy criteria beyond quality metrics, such as predictive accuracy. The second challenge was democratizing ML to allow biologists and clinicians to use ML tools. A key feature of TPOT is that it takes much of the guesswork out of ML by automatically exploring the ideal combinations of feature selectors, feature transformers, classification and regression methods, and hyperparameter settings. Automation and democratization of ML has the potential to accelerate biomedical research by reducing the time it takes to manually build and evaluate pipelines while at the same time expanding the user base.^1^^,^^2^^,^^15^

In this paper, we review TPOT and its applications in the medical domain, paying special attention to the latest version, TPOT2. There are three main goals for this paper:

(1) present an overview of the underlying algorithm driving TPOT,
(2) highlight the key differences between TPOT1 and TPOT2, and
(3) survey different domains where TPOT has been applied.

### Evolutionary pipeline optimization via GP

Evolutionary computation (EC) is a family of optimization algorithms inspired by natural selection and biological evolution that has demonstrated strong performance in tackling problems with infinite search spaces and no clear gradient toward an optimum.^30^^,^^31^ GP^27^ as one such method is a powerful population-based optimization technique where candidate solutions within a population undergo iterative evolution to address a given problem. Initially, GP was developed primarily to evolve functional computer programs,^27^ which are commonly represented as tree structures, similar to those found in programming languages like Lisp. The original use of tree structures to represent solutions in GP naturally facilitates the tree-like representations of ML pipelines, enabling a seamless adaptation of GP for AutoML. The TPOT^26^ is the first AutoML system to incorporate GP for ML pipeline optimization and provides an ideal platform for testing various theories and techniques within the field of EC to improve pipeline optimization.

Typically, GP begins by generating a set of randomly composed candidate solutions that form the starting population. The underlying representation of these solutions is predefined and specifically tailored to address the given problem. After constructing the initial population, each solution is evaluated with a set of user-defined fitness functions that measure its effectiveness in solving the given problem. A parent selection algorithm is then used to identify promising solutions that serve as parents for constructing offspring. The parent solutions provide genetic material that undergoes mutation, which modifies specific components of a solution, or crossover, which combines elements from two solutions to generate a new one. This process results in offspring solutions that inherit and potentially improve problem-solving capabilities. Once all offspring are constructed, they form a new population and undergo the same evolutionary process as their predecessors. This evolutionary cycle continues until a user-defined stopping criterion is satisfied, such as reaching the maximum number of generations or detecting no progress over a predefined period. A key difference between GP in AutoML and its application in tasks such as program synthesis lies in how solutions are evaluated. In AutoML, evaluation involves separate training and testing phases for each solution (pipeline), whereas in program synthesis, solutions are tested on a set of cases without a distinct training step. This distinction adds complexity when using GP in AutoML settings.

An effective AutoML optimization strategy should efficiently search and identify a promising ML pipeline within a reasonable time frame.^17^ The success of any such strategy largely depends on the trade-off it exhibits between exploitation and exploration of the search space. Exploitation enables the search to concentrate on high-performing pipelines, while exploration facilitates the discovery of unexplored regions within the pipeline search space. Indeed, a trade-off is necessary; relying solely on exploitation may collapse the search to only a limited set of high-performing pipelines, potentially missing out on other optima, while focusing exclusively on exploration can prevent the search from converging to optimal pipelines. GP provides multiple mechanisms to control this trade-off and has been extensively studied in the field of EC.^32^ For example, exploitation can be lowered by selecting parents that exhibit novel characteristics of interest beyond predictive performance, thereby generating offspring that explore less crowded regions of the search space (e.g., novelty search^33^). Conversely, exploration can be adjusted by varying the probability of mutations used to produce offspring: increasing this likelihood facilitates the exploration of distant neighboring pipelines, while decreasing it results in smaller, more localized searches within the neighboring search space.

### TPOT optimization strategy: Non-dominated sorting genetic algorithm II

TPOT uses the non-dominated sorting genetic algorithm II (NSGA-II)^34^ as its underlying framework for optimizing ML pipelines. NSGA-II is a multiobjective evolutionary algorithm designed to evolve a population of solutions that approximate the true Pareto front for a set of user-defined objectives. A Pareto front is a set where no solution in the set can be improved in one objective without worsening at least one other objective. Identifying a diverse set of Pareto-optimal solutions (i.e., the solutions on the Pareto front) is crucial for multiobjective problems, as these problems inherently involve trade-offs among competing objectives. For example, when purchasing a car with both safety and maximum speed as key considerations, the space of possible choices is vast. Prioritizing speed over safety would typically necessitate reducing the car’s weight, which, in turn, compromises its overall safety. At one extreme, the safest option might be an ambulance, while at the other extreme, the fastest could be a high-end luxury sports car. While both represent valid solutions, the most interesting trade-offs emerge from cars that balance these objectives. In this example, NSGA-II would be expected to return the complete set of Pareto-optimal solutions, analogous to the diverse range of cars that achieve varying balances between safety and speed.

A pseudocode describing the NSGA-II algorithm implemented within TPOT is given in Algorithm 1. The evolutionary search starts by initializing a population of randomly generated pipelines (line 2 in Algorithm 1). The structure of each pipeline is randomly assembled, incorporating a randomly selected ML method and a randomly assigned set of hyperparameters. These pipelines are evaluated on a set of user-defined objective functions (line 3 in Algorithm 1). Typically, TPOT optimizes two types of objectives: primary and secondary. The primary objectives optimize performance metrics, such as accuracy or precision, while the secondary objectives consider pipeline characteristics, such as pipeline complexity, in terms of the number of ML methods used. By default, TPOT uses *k*-fold cross-validation^35^^,^^36^^,^^37^ (with a user-defined *k*) when calculating objectives to reduce the risk of overfitting. Further modifications are required to use alternative performance metrics if desired. Each objective is used to generate a set of *k* scores; for each of the *k* folds, the pipeline is evaluated on that fold after training on the remaining folds. A single cross-validation score is calculated for a given objective by averaging all *k* scores across folds, providing the multiobjective performance assessment of a candidate pipeline.

Algorithm 1 **TPOT algorithm**

1: **procedure** TPOT(pop_size,max_gens)

2: population = InitializePopulation()

3: Evaluate(population)

4: NonDominatedScores(population)

5: **for** gen = 1 to max_gens **do**.

6:  parents = ParentSelection(population) ▷ Selected parents used to produce offspring

7:  offspring = GenerateOffspring(parents) ▷ Mutation and crossover applied to parents

8:  Evaluate(offspring)

9:  NonDominatedScores(population+offspring)

10:  population = SurvivalSelection(population, offspring, pop_size)

11: **Return** best-individual ▷ From all evaluated individuals

The cross-validation scores (one per objective) a pipeline receives are used to determine both the Pareto front rank and the crowding distance for each pipeline (line 4 in Algorithm 1; section III-B in Deb et al.^34^). The Pareto front ranking of a pipeline is determined by its Pareto optimality relative to all other pipelines in the population. The first (best) Pareto front is formed by selecting all nondominated solutions from the population; solution X is said to dominate solution Y if X is at least as good as Y across all objectives and is strictly better than Y in at least one objective. Subsequent fronts are constructed by selecting solutions that have not yet been assigned to a Pareto front, and this process continues iteratively until all solutions in the population are assigned to a front. The crowding distance of a pipeline is determined by evaluating its relative proximity to other pipelines within the same front across all objectives. Specifically, for each pipeline, we compute the distance between its two nearest neighbors (in the objective space) for each objective; the actual crowding distance is calculated as the average distance across all objectives. Larger distances are preferred, as they indicate that the pipeline occupies a less crowded region of the Pareto front, thereby promoting solution diversity.

The Pareto front rank and crowding distance assigned to a pipeline are used during the parent selection process (line 6 in Algorithm 1) to identify a set of parent pipelines that will contribute genetic material for offspring generation. By default, nondominated binary tournament selection is used to guide the population toward the true Pareto front by prioritizing pipelines with low front rankings and high crowding distance. Parents with low front rankings can generate offspring that potentially advance the current Pareto front toward the true front, while parents with high crowding distance can produce offspring that explore novel regions of the current Pareto front. This parent selection algorithm selects a parent pipeline by randomly sampling a pre-defined number of pipelines (by default, 2) to form a tournament, where the tournament winner is determined based on Pareto front ranking and crowding distance. Specifically, pipelines within a tournament that are not tied for the lowest ranking are discarded. Among the remaining pipelines, those that do not share the highest crowding distance are also removed. If multiple pipelines remain after both filtering events, one is randomly returned as a parent. The parent selection process also ensures that the appropriate number of parents is obtained to generate a sufficient number of offspring.

Once the required number of parents has been selected, offspring are generated by inheriting genetic material from these parents (line 7 in Algorithm 1). This genetic material undergoes variation, resulting in differences between the pipelines of the parents and their offspring. Typically, two types of variation operators are used to produce offspring: mutation and crossover. Mutation operators require only a single parent to generate offspring, applying probabilistic alterations to the offspring’s pipeline structure and its individual components, such as models or hyperparameters. Crossover operators, in contrast, require two parents to generate offspring by combining different sections of both parent pipelines to form a single offspring. Additionally, mutation can be applied to the offspring produced through crossover to introduce further variation, or parents can be mutated first before applying crossover to produce offspring. Both variation operators can contribute to the discovery of better pipelines, albeit to different extents. An offspring generated through mutation closely resembles its parent’s pipeline. On the other hand, an offspring produced via crossover inherits a combination of genetic material from both parents, thereby positioning the offspring between the two parent pipelines within the search space. Of course, the magnitude of these operators is also influenced by the probabilities (or “rates”) used; the higher these rates are, the more alterations the operators make to the parents to produce offspring.

After generating the offspring, they are evaluated in the same manner as their parents (line 8 in Algorithm 1), and assigned Pareto front rankings and crowding distances relative to both the current population and the newly generated offspring (line 9 in Algorithm 1). Survival selection is performed after the combined set of the current population and offspring has been assigned their Pareto front ranking and crowding distance (line 10 in Algorithm 1). Specifically, survival selection reduces this combined set of offspring and the current population back to the original population size by retaining the Pareto optimal pipelines. This selection process begins by selecting all pipelines that belong to the first (best) front. If the number of pipelines in this front exceeds the original population size, then those with a greater crowding distance are prioritized for survival. Conversely, if additional survivors are needed after processing the first front, pipelines from subsequent fronts are collected, again prioritizing those with a lower Pareto front rank and greater crowding distance. Once the complete set of surviving pipelines is formed, it constitutes the next generation and undergoes the same evolutionary cycle for a predefined number of generations (line 5 in Algorithm 1). Upon completion of an evolutionary run, the optimal pipeline is returned, which the users can export for further investigation or deployment within their specific application domain (line 11 in Algorithm 1).

With respect to runtime, there are three primary categories of parameters that directly influence the total execution time of a run. From the perspective of GP parameters, both the population size and the number of generations significantly affect runtime, as their product determines the total number of pipelines evaluated. Additionally, the complexity of the pipeline search space impacts runtime, since more complex models generally require longer evaluation periods. Finally, the user may specify a time constraint for individual pipeline evaluations; in the absence of such a limit, the evaluation proceeds until completion.

The evolutionary processes described above offer an effective method for systematically evaluating more pipeline configurations than would be possible with manual design and tuning. Additionally, it allows the simultaneous optimization of multiple criteria when searching for effective pipelines, which could be a requirement for biomedical domains. Consequently, NSGA-II serves as a robust strategy for automated pipeline optimization.

### Comparison of TPOT variants

The two main iterations of the TPOT package, TPOT1^26^ and TPOT2,^21^ differ primarily in the type of representation used for evolving ML pipelines. However, the underlying GP algorithm remains unchanged. The specifics of both iterations are discussed in the following sections.

### TPOT1 specifics and limitations

The initial implementation of TPOT optimizes ML pipelines using a tree-based representation, aligning with the meaning of its acronym.^26^ Each pipeline is represented as an expression tree, where internal nodes denote ML operators, and leaf nodes correspond to the hyperparameters associated with their respective internal nodes. Through iterative evolution using selection, crossover, and mutation, TPOT1 explores a diverse pipeline search space to optimize ML workflows and discover high-performing pipelines. The nodes within a given pipeline are categorized into three types.

(1) Leaf nodes represent the hyperparameters associated with a particular ML operator node (e.g., the number of trees in a random forest classifier).
(2) Inner nodes contain a particular ML operator (e.g., standard scaler or principal-component analysis).
(3) root node holds the final ML classification or regression model (e.g., linear regression or random forest classifier).

The ML operators used within TPOT1 fall into one of three main categories^38^: feature preprocessors (modify the features), feature selectors (select some features), and classification or regression methods. All ML operators are implemented using existing components from scikit-learn.^39^

The overall pipeline workflow in TPOT1 follows a tree-based structure, ensuring that every node is connected, no cycles are present within the pipeline, and all paths ultimately converge at the root node. All pipelines begin by processing a dataset, which is then passed to the initial set of ML operators. These operators belong to one of three categories described previously, determining their specific role within the pipeline. In the simplest scenario, the data are directly provided to the root node for classification or regression. However, more complex pipeline architectures may incorporate multiple feature engineering and feature selection processes, each selecting and transforming the data differently. In TPOT1, often, multiple copies of the input dataset are generated and processed independently through these stages. Each copy can undergo multiple different feature transformations, selections, and engineering steps. The newly generated and selected features are then merged through a combination operator, which combines features from different copies while making sure there are no duplicate features. The resulting dataset is then passed to the final classifier or regressor for training.

**Figure 1:**
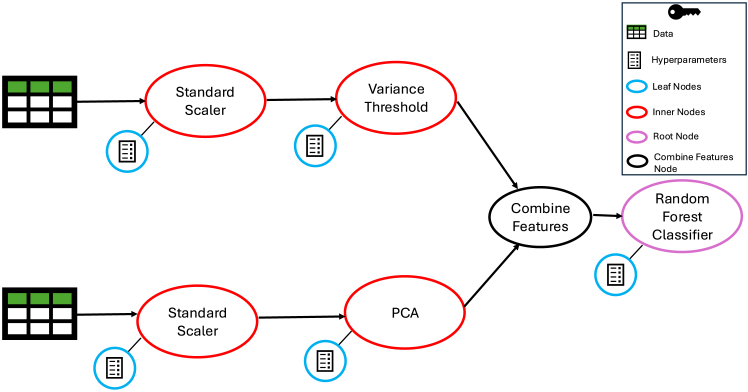
Tree-based representation of an ML pipeline in TPOT1

Figure 1 illustrates an ML pipeline using five unique operators: standard scaler (feature transformation), variance threshold (feature selection), combine features (feature transformation), and principal-component analysis (PCA) (feature transformation). One dataset copy undergoes standard scaler and variance threshold, and a copy of the same dataset passes through standard scaler and PCA. Their outputs are merged via the combine features operator and fed into a random forest classifier. This TPOT1 pipeline representation highlights three key limitations. First, the output of a single node cannot be shared with multiple nodes. This can limit the propagation of useful information in the pipeline. For example, if one node is able to construct some useful features, then it will not be able to pass these features to more than one node for further processing. Second, the tree structure can lead to duplicated effort. If one particular feature transformer is needed in two different paths, then the same operator needs to be applied twice. Third, as the pipelines grow more complex, the tree structure can become less space efficient because of the need to store multiple copies of ML operations and transformed data.

TPOT1 leverages the Distributed Evolutionary Algorithms in Python (DEAP)^40^ package for evolutionary optimization, using its core functions for mutation, crossover, reproduction, evaluation, and selection. Within TPOT1, DEAP is configured to optimize both the sequence of pipeline operators and their corresponding hyperparameters to identify the best-performing pipeline with the NSGA-II algorithm (Algorithm 1). NSGA-II maintains a Pareto front of optimal solutions across generations, optimizing two competing objectives: pipeline predictive performance (accuracy, precision, etc.) and pipeline complexity (measured as the total pipeline operators). At the end of an evolutionary run, TPOT1 returns the best-performing pipeline. Using DEAP offers both advantages and limitations. On one hand, it offers a robust evolutionary framework for evolving GP-based ML pipelines with readily available implementations, thereby simplifying development. However, this predefined framework restricts flexibility, particularly when deviating from TPOT1’s original implementation; for instance, integrating more advanced tools for parallelization and data management or enabling finer-grained control over the execution of evolutionary processes. For example, users who require custom variation operators for specific nodes will not be able to implement these operators within DEAP.

Although the initial population of pipelines is typically generated randomly, this may not always be the preferred approach. TPOT1 allows users to define a custom pipeline structure using a “template” parameter. By default, TPOT1 constructs pipeline structures randomly; however, when a template is provided, it enforces a predefined linear sequence of ML operators, ensuring a more controlled evolutionary process. Each step in the template must belong to one of four classes: selector, transformer, classifier, or regressor. Users can also specify a broad category (e.g., “transformer”), allowing TPOT1 to randomly select an operator from that class, or a specific operator (e.g., “LinearRegression”) from TPOT1’s configuration. This flexibility enables users to tailor pipeline structures based on their application domain and modeling requirements while still leveraging TPOT1’s automated optimization.

Several extensions of TPOT1 have been developed to address specific challenges and expand its capabilities in AutoML. TPOT-NN^41^ integrates neural network estimators into TPOT1’s pipeline search, enabling AutoML augmentation with deep learning models. While TPOT-NN has shown performance comparable with or superior to standard TPOT1 in some cases, it requires longer training times. This extension paves the way for future research into more complex AutoML architectures. TPOT-FSS^42^ improves TPOT1’s efficiency for high-dimensional datasets. First, the features are partitioned into multiple smaller subsets. Then, the GP algorithm selects the most relevant subsets for the final pipeline. This increases TPOT1’s scalability, making it more suitable for big data applications, such as RNA sequencing analysis. TPOT-MDR^43^ integrates TPOT1 with the multifactor dimensionality reduction (MDR)^44^ algorithm, which models higher-order feature interactions. Tailored for genetic analysis, this extension incorporates MDR-based feature construction and expert knowledge-guided feature selection, making it particularly useful for bioinformatics and biomedical research.

### TPOT2 specifics and upgrades

To enhance modularity and extensibility across diverse application domains, TPOT2^21^ was developed as an improved version of TPOT1. While the core principle of AutoML via GP remains unchanged, TPOT2 replaces the tree-based pipeline representation of its predecessor with a graph-based structure, enabling greater flexibility and expressiveness in pipeline design. In TPOT2, each ML pipeline is modeled as a directed acyclic graph (DAG), implemented using NetworkX^45^ Python package. Unlike TPOT1, where ML operators are represented as internal nodes and hyperparameters as leaf nodes, TPOT2 integrates ML operators and their corresponding hyperparameters within a single node, streamlining the uniform representation of nodes in pipelines. The TPOT2 graph structure consists of three main node types.

(1) Leaf nodes represent ML transformers or selectors, including their hyperparameters, that directly receive raw data as input and transmit processed features to inner nodes.
(2) Inner nodes represent ML transformers or selectors, including their hyperparameters, that process outputs from leaf nodes or other inner nodes and pass the transformed data to subsequent inner nodes or the root node.
(3) The root node represents the final ML classifier or regressor model, including its hyperparameters, which receives inputs from inner and leaf nodes to generate the final predictions.

Since TPOT2 uses a DAG representation, the directionality of the pipeline structure defines the flow of information to successive nodes. Similar to TPOT1, all ML functions and methods in TPOT2 are implemented using scikit-learn.^39^

**Figure 2:**
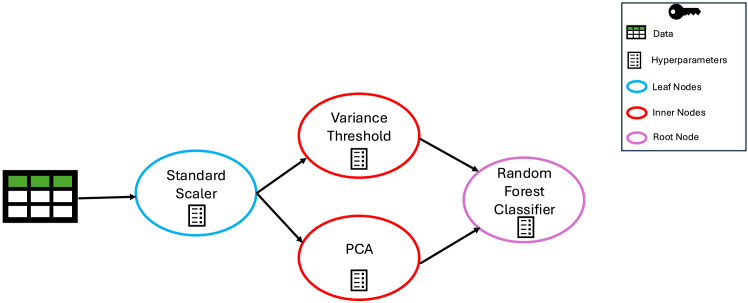
Directed acyclic graph representation of an ML pipeline in TPOT2

The graph-based representation in TPOT2 allows for more flexible and realistic ML workflows by enabling multiple data transformation and selection steps to be reused and dynamically connected. This modular design better reflects real-world ML pipelines and expands the range of possible solutions. Additionally, integrating ML operators with their hyperparameters ensures more efficient optimization by making it possible to simultaneously change the model as well as its hyperparameters. Overall, transitioning from a tree-based to a DAG-based structure enhances flexibility, efficiency, and expressiveness, making TPOT2 better suited for high-dimensional data, complex model architectures, and diverse AutoML applications. We can see how the structure representing the same pipeline is more intuitive with TPOT2’s representation, as Figure 2 shows the same ML pipeline as Figure 1 but with TPOT2’s DAG representation. In Figure 2, a single copy of the dataset is passed to the standard scaler, whose output is simultaneously sent to both variance threshold and PCA, simplifying output sharing across multiple nodes. Finally, their outputs are fed directly into the random forest classifier, eliminating the need for the combine features operator required in TPOT1.

A key advancement in TPOT2 is the transition from the DEAP^40^ package to a custom-built evolutionary algorithm module, providing greater flexibility, extensibility, and control over the optimization process. Specifically, the NSGA-II algorithm, outlined in Algorithm 1, was implemented from scratch to provide finer control over the evolutionary process and its underlying intricacies. In TPOT1, attempting to deviate from its original implementation or attempting to use custom evolutionary procedures would require directly modifying the core source code. However, the custom-built NSGA-II algorithm in TPOT2 supports user-defined variation functions, enabling more tailored evolutionary strategies. Additionally, it allows seamless integration of new optimization and evaluation functions, while different selection algorithms can be specified as parameters, enhancing adaptability and fostering greater experimentation.

Similar to the concept of templates in TPOT1, TPOT2 introduces search spaces. A search space defines possible pipeline structures, including the ML methods and hyperparameter ranges for each step. An example search space (simplified for illustrative purpose) could be SequentialPipeline([“selectors”, RandomForestClassifier]). This search space constraints the pipelines to choose one of the available feature selection operators followed by a random forest classifier model. TPOT2 provides many built-in search spaces: SequentialPipeline (fixed length linear pipelines), DynamicLinearPipeline (variable length linear pipelines), tree, graph, etc. Additionally, users can define custom search spaces tailored to specific domain tasks.

Like its predecessor, TPOT2 remains easily extendable for specialized tasks, such as integrating neural networks. Its modular design ensures that components can be efficiently customized or replaced. TPOT2 leverages Dask (https://github.com/dask/dask) for parallel execution, maintaining scalability for large-scale ML workflows.

### Overcoming TPOT challenges

While TPOT provides an effective framework for the automated discovery of ML pipelines, it is not without limitations. As with many AutoML systems, the computational cost associated with identifying optimal pipelines remains a significant concern. Users must account for both the total number of pipelines evaluated during a run and the cost of evaluating each pipeline. For instance, evaluating a linear regression model is generally less computationally demanding than evaluating a neural network. TPOT incorporates several mechanisms to mitigate these costs, including an early stopping criterion that terminates the search when insufficient improvement is observed over a period of time. In the worst-case scenario, users can reduce the number of pipelines evaluated by decreasing the number of generations, the population size, or both.

Another issue for TPOT is that the complexity of the returned model may be too high, resulting in a loss of interpretability. For example, the final pipelines output by TPOT can sometimes be very complex, such as a neural network model being returned when a linear regression could have been sufficient. One workaround for that is to use complexity as one of the objectives during TPOT optimization, as is done regularly in the case of TPOT2. Moreover, users can customize the search space by excluding computationally expensive models and operators or by imposing constraints on the pipeline structure to further reduce the computational burden.

Although TPOT can be used in an out-of-the-box setting, more often than not, the user needs manual tuning when it comes to determining the GP parameters like population size, the maximum number of generations, and other parameters, like the objective functions to be used. Furthermore, like many other software packages, TPOT can become increasingly cumbersome over time due to the accumulation of customizations that are not enabled by default. This added complexity can hinder users who wish to modify or extend the underlying software.

### Applications of TPOT

TPOT has been widely applied across various biomedical domains, particularly for disease diagnosis and outcome prediction based on patient data. The application of TPOT spans a variety of medical conditions and imaging modalities, demonstrating its versatility in AutoML pipelines for clinical decision support. Ultimately, TPOT demonstrates its ability to tackle the complexities of medical and health applications through evolutionary AutoML.

Developing ML pipelines capable of accurately predicting disease diagnoses or adverse medical outcomes is a significant objective for researchers. Accurate disease diagnosis can serve as a valuable second opinion for healthcare providers, while precise prediction of adverse outcomes can help mitigate the risk of future negative events. Regarding disease diagnosis, TPOT has been used to identify major depressive disorder,^42^ coronary artery disease,^46^ hepatocellular carcinoma,^47^ endometrial cancer,^48^ and breast cancer.^49^ It has also been applied to detect myocardial fibrosis,^50^ lung cancer from computed tomography scans^51^^,^^52^, and gliomas—malignant brain tumors—using radiomics and other imaging features.^53^^,^^54^^,^^55^^,^^56^ Furthermore, TPOT has been leveraged for diagnosing atherosclerosis, a condition involving fatty deposits in arterial walls,^57^ and for detecting renal cell carcinoma based on radiomics features.^58^ Beyond diagnosis, TPOT has been instrumental in predicting adverse medical outcomes. For instance, it has been used to assess risks following spinal surgery,^59^ forecast functional outcomes for patients undergoing mechanical thrombectomy (a procedure for removing arterial blood clots),^60^ and predict liver damage caused by hepatotoxins.^61^ It has also facilitated the prediction of adverse drug events in both older adults^62^ and pediatric patients.^63^ Additionally, it has played a role in environmental and toxicological studies, such as predicting chemically induced disruptions to estrogen, androgen, and thyroid hormone modalities.^64^ Other applications include analyzing fetal health^65^ and detecting cerebral cystic metastases.^66^

Genetic datasets are typically high dimensional, often containing hundreds of thousands to millions of features, such as single-nucleotide polymorphisms (SNPs), gene expression levels, or epigenetic markers. Effectively analyzing such data requires robust feature selection and model optimization techniques. TPOT is well suited for these challenges for two main reasons: it integrates automated feature selection, and ML operators used by TPOT can be customized to address specific genetic analysis tasks. TPOT has, therefore, found success when used in genetic analysis. For example, in Freda et al.,^67^ the authors modified the ML operators in TPOT, specifically feature selection and transformation methods, in order to use it to come up with a pipeline for quantitative trait locus analysis and epistasis exploration. Tejera et al.^68^ predicted, using TPOT, whether a given protein is a target or off target, helping identify potential drug candidates for preeclampsia by evaluating new compounds based on the trained ML models. Bonnidia et al.^69^ used TPOT to predict non-coding RNAs in bacteria. Manduch et al.^70^ used TPOT in the identification of genetic pathways and genes associated with creatinine levels in rat kidneys.

While TPOT has mostly been used in biomedical settings, its use in non-clinical domains has been increasing over the years. For example, it has been employed to detect distributed denial of service attacks in the cybersecurity domain,^71^ predict the concentration of *E. coli* in drinking water,^72^ classify real cases of whistleblowing of academic dishonesty,^73^ predict the amount of methane (working capacity) that can be stored within a covalent organic framework material at specific conditions,^74^ predict fabric quality in the textile manufacturing sector from the information derived from sensors embedded in textile machinery,^75^ model how different properties of biochar (a form of charcoal made from organic waste or biomass) influence anaerobic digestion efficiency,^76^ classify an area infected with an invasive plant species from satellite images,^77^ predict the air quality index from environmental data,^78^ and predict the properties of steel in a task called material mechanical property prediction used during steel production.^79^

### Future directions

Although TPOT has demonstrated effectiveness as an AutoML tool across various domains, there is still room for further improvement. Notably, while TPOT is the first AutoML system to use GP as its core optimization strategy for ML pipeline optimization, the broader field of AutoML is a relatively recent development and is a rapidly evolving domain with continuous advancements. In contrast, the field of EC has a longer history and provides numerous promising techniques and concepts that have yet to be fully explored within the AutoML domain. For example, the field of GP has investigated a variety of evolutionary operators that influence the probability of a successful evolutionary run, including variation operators, population structures, fitness functions, and selection mechanisms. A straightforward improvement to TPOT could involve updating its core optimization strategy to a more modern approach, such as integrating the next version of the NSGA, specifically NSGA-III.^80^^,^^81^^,^^82^ Future work could investigate the impact of incorporating NSGA-III on TPOT’s effectiveness, particularly in scenarios where obtaining a well-defined Pareto front of ML pipelines is crucial for success, such as health and medical domains.

The optimization strategy underlying TPOT uses a multiobjective approach to generate a set of ML pipelines that uniquely balance trade-offs between conflicting objectives. However, not all ML problems or tasks necessarily require multiple objectives to identify optimal pipelines. If the pipeline search space is defined so that complexity is constrained within a desired range, optimizing solely for accuracy may be sufficient to identify optimal pipelines, eliminating the need to explicitly optimize for both pipeline complexity and predictive performance. The incorporation of additional information into a single objective has been investigated in EC, exemplified by fitness sharing,^83^ which adjusts a solution’s performance by applying a penalty based on the density of solutions within the genotypic or phenotypic space. Alternatively, the decomposition of a single-valued performance metric has also been investigated within EC, notably with the development of lexicase selection,^84^ particularly in the context of program synthesis using GP. Lexicase selection is a parent selection method that identifies parents based on their performance on individual test cases, in contrast to the traditional approach of aggregating test case results into a single performance metric (e.g., accuracy). In fact, lexicase selection inspired the development of lexidate,^85^ a parent selection method evaluated within TPOT to identify parents based on their predictive performance on individual data samples. Future work can investigate how to best optimize pipelines by working with only one objective.

Large language models (LLMs) offer several new directions for TPOT research. First, the ability to interact with TPOT using natural language can potentially further expand the base of users by eliminating the need to write code or scripts to launch analyses. The use of LLMs in this regard will further democratize the approach, making it much more user friendly. Second, LLMs can be used to query knowledge bases, such as the AlzKb^86^ knowledge graph, which contains biomedical knowledge for Alzheimer’s disease. This capability enables the integration of domain-specific insights into ML tasks, including feature selection and model interpretation. In fact, Elisabetta et al.^87^ used TPOT to identify combinations of SNPs associated with coronary artery disease. To achieve this, they leveraged Hetionet, a publicly available biomedical database, to group SNPs into biologically meaningful sets, which were then used by TPOT’s feature selection operators. Finally, LLMs could be designed and used to query a database of TPOT results to allow for natural language search and interpretation of results. This will further expand the democratization of ML. LLMs and other computational methods for interacting with TPOT could be implemented as part of an agentic approach with specific LLM agents designed for specific tasks such as feature selection, TPOT pipeline specification, and biological interpretation. Modern AI tools will significantly enhance the development, application, and deployment of AutoML tools such as TPOT.

Incorporating interpretability and explainability into AutoML pipelines is crucial to ensuring that models are not only accurate but also transparent. This is particularly important in applications like disease prognosis, where understanding the reasoning behind a model’s predictions can aid clinical decision-making. Currently, TPOT users typically apply explainability tools like Shapley additive explanations^88^ and local interpretable model-agnostic explanations^89^ after identifying the best model. A future direction for TPOT could involve integrating these interpretability methods directly into the pipeline, providing users with insights into how data transformations, feature selection, and individual features contribute to the final prediction. This would enhance transparency and usability, making TPOT’s outputs more informative and actionable. Ensuring fairness, privacy, and adaptability in AutoML is also becoming increasingly important, particularly in sensitive domains like healthcare. ML models trained on real-world data can sometimes inadvertently make biased predictions that negatively impact marginalized communities. This makes it crucial to integrate fairness-aware optimization techniques into TPOT. Additionally, privacy-preserving methods, such as federated learning, could enable TPOT to train models across decentralized datasets without directly sharing sensitive data, ensuring compliance with data protection regulations. Future enhancements to TPOT could incorporate bias-mitigation strategies and federated learning capabilities, making it more ethical, secure, and applicable to real-world scenarios involving distributed and privacy-sensitive data.

## Resource availability

### Lead contact

Requests for further information and resources should be directed to and will be fulfilled by the lead contact, Jason H. Moore (jason.moore@csmc.edu).

### Materials availability

This study did not generate new unique reagents.

### Data and code availability

• The original source code for TPOT1 can be found at https://github.com/EpistasisLab/tpot/tree/master-archived.
• The Original source code for TPOT2 can be found at https://github.com/EpistasisLab/tpot.

## Acknowledgments

This work was funded by 10.13039/100000002National Institutes of Health grants LM010098, LM014572, and AG066833. The authors thank all members of the Department of Computational Biomedicine at 10.13039/100013015Cedars-Sinai Medical Center for their support.

## Author contributions

Conceptualization, J.G.H. and A.K.S.; TPOT1 specifics, A.G.; TPOT2 specifics, J.G.H. and A.G.; applications of TPOT, A.K.S. and A.G.; writing – original draft, J.G.H., A.K.S., and A.G.; writing – review & editing, J.G.H., A.K.S., A.G., and J.H.M.; funding acquisition, J.H.M.; supervision, J.H.M.

## Declaration of interests

J.H.M. is part of the *Patterns* advisory board.

## Declaration of generative AI and AI-Assisted technologies

During the preparation of this work, the author(s) used ChatGPT-4o to refine sentences only for clarity. After using this tool or service, the author(s) reviewed and edited the content as needed and take(s) full responsibility for the content of the publication.
